# Magnetism and Luminescence of a MOF with Linear Mn_3_ Nodes Derived from an Emissive Terthiophene-Based Imidazole Linker †

**DOI:** 10.3390/molecules26144286

**Published:** 2021-07-15

**Authors:** Weiran Wang, Junpeng He, Hongyu Guo, Samuel G. Dunning, Simon M. Humphrey, Richard A. Jones

**Affiliations:** Department of Chemistry, The University of Texas at Austin, 105 E. 24th Street, Stop A5300, Austin, TX 78712-1224, USA; wrwang@utexas.edu (W.W.); hjy91523@163.com (J.H.); hongyuguo@utexas.edu (H.G.); sdunning@carnegiescience.edu (S.G.D.)

**Keywords:** terthiophene, imidazole, metal-organic frameworks

## Abstract

A new terthiophene-based imidazole luminophore 5,5’-(1*H*-thieno[3,4-d]imidazole-4,6-diyl)bis(thiophene-2-carboxylic acid) (TIBTCH_2_, **5**) was synthesized in one step from previously reported 4,6-di(thiophen-2-yl)-1*H*-thieno[3,4-d]imidazole (DTTI, **4**), and their photophysical properties were studied and compared accordingly. Under solvothermal conditions, reacting **5** with Mn(OAc)_2_ yielded a new three-dimensional metal-organic framework (MOF, **6**) which was structurally defined by single-crystal X-ray diffraction. In **6**, all Mn(II) ions octahedrally bind to carboxylate-*O* atoms to form a linear Mn_3_ secondary building unit (SBU) that contains three distinct coordination modes. Importantly, **6** exhibits dual functional properties of ligand-based emission and metal-based magnetic behaviors.

## 1. Introduction

Metal-organic frameworks (MOFs) are porous crystalline materials constructed with metal ions or clusters as secondary building units (SBUs) joined into infinite arrays by organic linkers. Emissive MOFs have been extensively studied as luminescent materials and sensors [1,2,3,4]. MOFs that are both emissive and display permanent porosity with large surface areas have been utilized to adsorb and detect various guest molecules, which can result in significant emission changes [4]. There is considerable current interest in the synthesis of emissive MOFs using customizable organic linkers and different metal SBUs driven by the goal of expanding the sensing applications of the MOF, as well as incorporating more diverse functional properties (e.g., magnetism [5] or conductivity [6,7,8]). Strategies for the design of emissive MOFs tend to rely upon either the inclusion of the emissive metals as the nodes or by incorporating organic chromophores into the ligand scaffold. MOFs with metal-based light emission can be utilized in molecular sensing by exploiting ligand-to-metal charge-transfer (LMCT) and by employing lanthanide (Ln(III)) metal centers coupled to organic “antennas”. Specifically, Ln(III)-based emissive MOFs harvest energy from the excited state of the organic linkers, which undergo internal resonant transfer to nearby Ln(III) centers, resulting in Ln-based emission. Short-range vibrational coupling of certain chemical bonds presented by guest adsorbates (e.g., C-H, N-H and O-H) with the Ln(III) centers can quench the luminescence via non-radiative decay processes to varying degrees [3]. As such, in optimal instances, the identity and concentration of adsorbed chemical species may be identified based on the resulting emission behavior of a MOF, thereby enabling quantitative analyte detection. Ligand-based emissive MOFs are often designed with rigid π-conjugated emissive organic linkers, which can be further classified into two categories: (1) emissive dicarboxylic acids linkers or (2) emissive N-containing linkers. MOFs based on emissive N-heterocyclic ligands have been demonstrated for sensing different analytes [1,4]. There appear to be fewer reports of emissive dicarboxylic organic linkers used in the design of emissive MOFs. Examples include 4,4′,4″-nitrilotrisbenzoic acid (H_3_NTB), [9] *trans*-4,4′-stilbene dicarboxylic acids [10,11], and 4,4′-ethyne-1,2-diyldibenzoate [12] as intrinsic emissive linkers; *tetrakis*(4-carboxyphenyl) ethylene [13] has been employed as an aggregation-induced emissive linker. Our studies have focused on expanding the family of emissive MOFs through the design of a new emissive dicarboxylic linker with the aim of enabling emission in the most well-studied MOF topologies based on linear (1,4-benzene) dicarboxylate linkers (e.g., MOF-5 [14,15] and UiO-66 [16]).

**Scheme 1:**
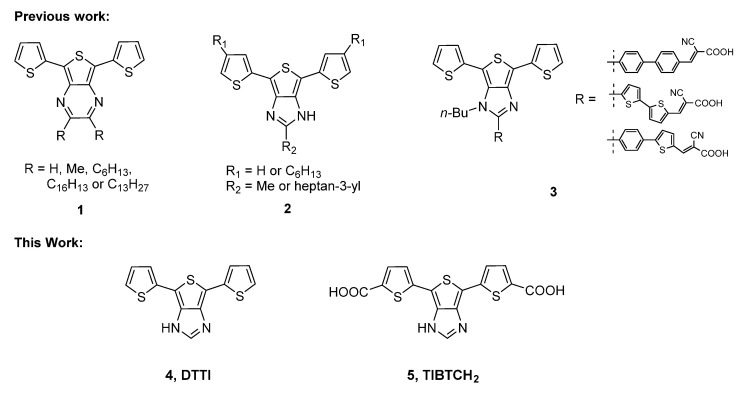
Different terthiophene-based *N-*fused heterocyclic (pyrazine or imidazole) molecules.

Terthiophene-based N-fused heterocyclic molecules such as 5,7-di(2-thienyl)thieno[3,4-b]pyrazine derivatives (**1**) are of interest as monomers for the synthesis of narrow band-gap conductive organic polymers [5]. Of related interest, 2-alkyl-4,6-di(2-thienyl)thieno[3,4-b]imidazole derivatives (**2**) have been used as the acceptor monomer unit in a series of donor-acceptor based organic solar cells [17]. Similarly, 2-aryl-4,6-di(2-thienyl)thieno[3,4-b]imidazole derivatives (**3**) have been studied as the electron donor species in dye-sensitized solar cells [18]. As part of a wider study of terthiophene-based imidazole molecules, we recently reported the preparation of 4,6-di(2-thienyl)thieno[3,4-b]imidazole (**4**, DTTI) [19] (Scheme 1). An easily prepared derivative of **4** is the dicarboxylic acid derivative, 5,5′-1*H*-thieno[3,4-d]imidazole-4,6-diyl)*bis*(thiophene-2-carboxylic acid) (**5**, TIBTCH_2_); here, we present an investigation of its efficacy in the construction of new emissive MOFs. Once functionalized with dicarboxylic acids, **5** (TIBTCH_2_) still possesses interesting photoluminescent properties; therefore, MOFs based on this linker were expected to possess functionalities stemming from both the organic linker and the metal SBUs. Herein, we describe the dual solid-state functions of a novel material, **6**, featuring terthiophene-based imidazole emissive linkers with linear Mn_3_ SBUs. Material **6** is a porous crystalline material with both emissive and magnetic functionalities.

## 2. Results and Discussion

### 2.1. Synthesis and Photophysical Studies of New Luminophores

**Figure 1:**
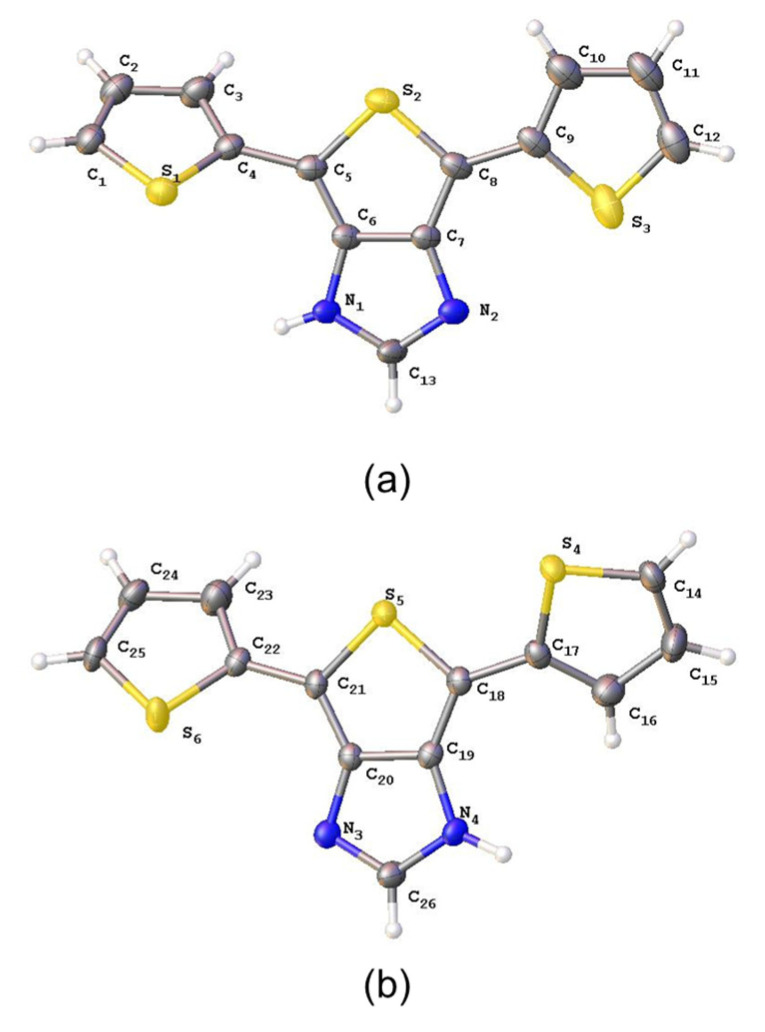
ORTEP views of the two molecules ((**a**) and (**b**), respectively) of **4** found in the asymmetric unit showing the atom labeling scheme. Displacement ellipsoids are scaled to the 50% probability level. The minor components of the disordered thiophene rings were omitted for clarity.

**Scheme 2:**
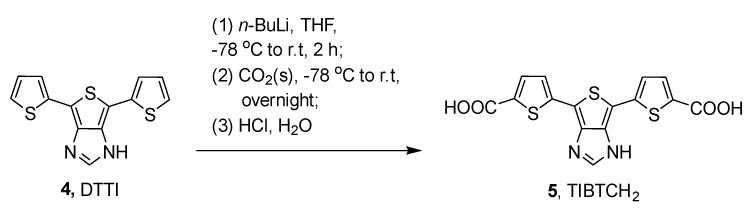
Synthesis of the ligand TIBTCH_2_ (**5**).

The parent terthiophene 4,6-di(2-thienyl)thieno[3,4-b]imidazole (**4**, DTTI) was prepared by following the previously reported method by our group [19]. Yellow needle-like crystals suitable for single crystal X-ray diffraction studies were collected after recrystallization from hot toluene/hexane (Figure 1). The compound crystallizes in the monoclinic space group, P2_1_/n (Z = 8) with two unique molecules in the asymmetric unit. A single toluene molecule resides on the 2-fold inversion center. In both molecules, the terminal thiophene units are disordered by approximately 180°, indicating free rotation of these units. These features are similar to those previously reported in other terthiophene structures [20]. Close-range intermolecular H-bonding contacts were also found between N1–H···N3 (2.088(0) Å) and N4–H···N2 (1.962(0) Å). Although N2 and N3 are expected to be basic, no hydrogens were found linking to them, based on crystallographic data and previously reported NMR spectroscopic data [19]. The dicarboxylic acid derivative **5** (TIBTCH_2_) could be synthesized by direct lithiation with 3.5 equivalents of n-BuLi (one sacrificial equivalent of n-BuLi reacts with the N–H proton of the imidazole moiety), followed by exchange with solid CO_2_, to yield the target material in good yield (Scheme 2). We were unable to obtain crystals of **5** suitable for single-crystal X-ray diffraction studies due to its low solubility in common solvents. 

**Figure 2:**
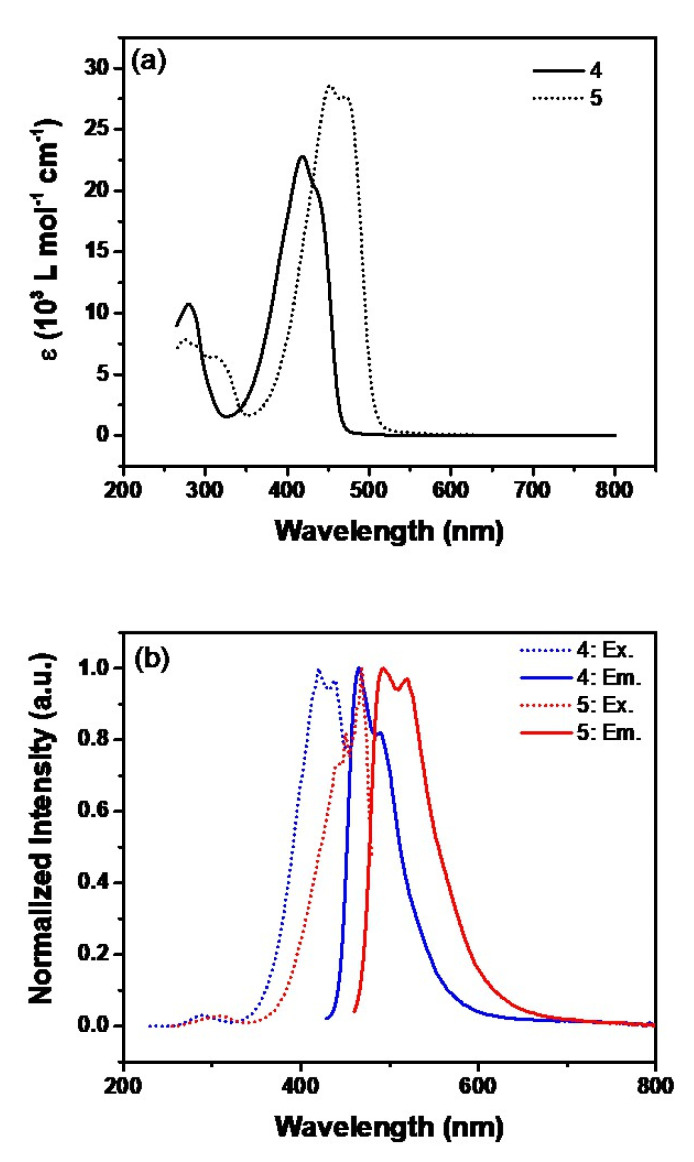
UV-Vis spectra (**a**) and normalized excitation and emission profiles (**b**) of **4** and **5** in DMSO (3.5 µM and 3.4 µM, respectively).

The photophysical properties of **4** and **5** were first studied in dimethyl sulfoxide (DMSO) at room temperature (Figure 2). The UV-vis absorption spectrum of **4** in DMSO revealed two absorption bands at 418 nm and 435 nm. A bathochromic shift of these bands is expected for **5** since it contains two electron-withdrawing carboxylic acid groups and the bands are observed at 453 nm and 472 nm. Both **4** and **5** exhibited strong observable photoluminescence even under daylight illumination. Molecule **4** showed two emission bands at 465 nm and 489 nm, and those of **5** were bathochromically shifted to 494 nm and 515 nm. 

### 2.2. Synthesis and Characterization of ***6***

**Figure 3:**
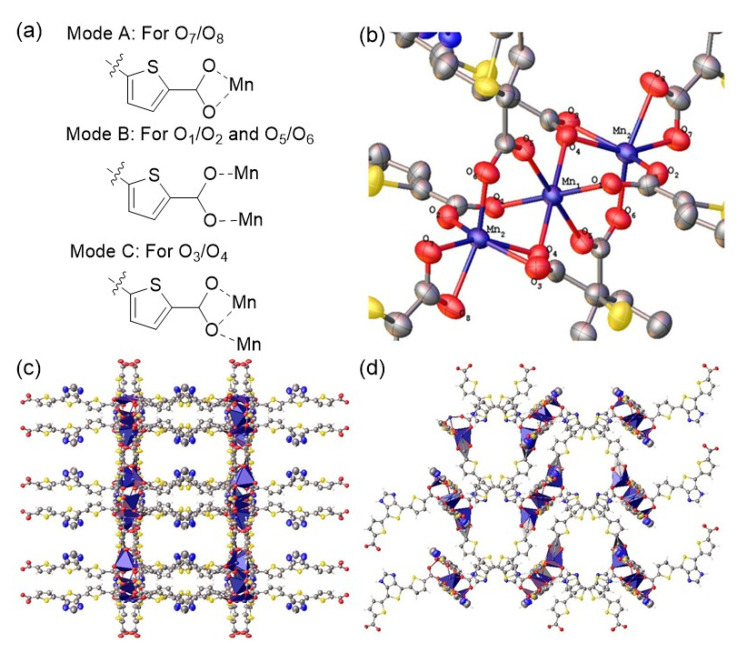
(**a**). Coordination modes A, B and C. Mode-A: 1,3-chelating; Mode-B: *syn,syn*-bridging; Mode-C: *syn, syn*-bridged with an additional 1,1’-single atom-*O* bridge. (**b**). Mn_3_ SBU of **6**. (**c**,**d**). crystal packing views along the crystallographic *a-* and *b*-axes, respectively. Anisotropic displacement ellipsoids are scaled to the 50% probability level. Hydrogen atoms of the structure were omitted for clarity for (**b**).

The dicarboxylic acid derivative **5** was used as a building block (linker) for the formation of a Mn(II)-based MOF (**6**). The new MOF, with empirical formula 2H·[Mn_3_(TIBTC)_4_]·3H_2_O, was obtained as a red needle-like polycrystalline material by treating a suspension of **5** (0.04 mmol) with Mn(OAc)_2_·4H_2_O (0.1 mmol) in a solution of DMF containing H_2_O (10 vol%) at 85 °C for 10 days. The atomic structure was determined by a single-crystal X-ray diffraction analysis, and the composition was confirmed by elemental analysis ((C_60_H_32_Mn_3_N_8_O_19_S_12_): Calcd. C, 41.94%; H, 1.88%; N, 6.52%; S, 22.39%. Found. C, 41.96%; H, 1.41%; N, 6.52%; S, 22.46%). Material **6** crystallizes in the orthorhombic Pcca space group and has an infinite 3-D structure comprising linear [Mn_3_]^6+^ units as the SBUs (Figure 3). The +2 oxidation state assignments for Mn1 and Mn2 in the asymmetric unit were established using the bond valence sum (BVS) method [21] (Table S1). We also utilized X-ray photoelectron spectroscopy (XPS) to confirm the oxidation state of Mn_3_ units in **6** by examination of the binding energy differences of the two 2s responses of each of the Mn atoms. Figure S1 shows the deconvoluted Mn 2s XPS spectra of as-synthesized **6** polycrystals. A 6.1 eV binding energy difference between two 2s peaks was observed. This value falls into the range of previously reported Mn(II) compounds (5.8–6.1 eV) and is significantly larger than the range of both Mn(III) (4.6–5.4 eV) and Mn(IV) (4.5–4.7 eV) compounds [22]. This data further supports the proposal that both Mn1 and Mn2 possess a +2 oxidation state in **6**.

Within each linear Mn_3_ SBU, the Mn1-Mn2 distance is 3.559(0) Å (Mn1 resides on an inversion center). This distance is longer than the previously reported average covalent diameter (twice the radii) of either high spin (h.s.) or low spin (l.s.) Mn ions (h.s. Mn: 3.22 Å; l.s Mn: 2.78 Å) [23], suggesting that there are no direct orbital–orbital interactions (i.e., non-bonding) between Mn1 and Mn2. The two symmetrically unique Mn(II) atoms Mn1 and Mn2 in each SBU have distinct coordination environments (Figure 3a,b)). The central Mn1 atom has an octahedral geometry with O1 and O5 from mode B and two bridging O atoms (O4) by mode C. The two terminal Mn2 atoms in each SBU have distorted octahedral geometries with O7 and O8 from mode A, O2 and O6 from mode B, and O3 and bridging O4 from mode C. These features are similar to those found in other reported MOFs with linear Mn_3_ SBUs [24,25]. Of the four crystallographically unique TIBTC^2−^ linkers, two of them connect to different SBUs in a “head-to-head” style, in which the terthiophene moieties of each organic linker point towards the other (Figure 3c,d). The remaining two TIBTC^2−^ linkers connect to Mn2 through its axial position on one end with another end bridging to Mn1 and Mn2 of a different Mn_3_ SBU. These coordination features, along with the curved terthiophene backbone of TIBTC, help to build a 3-D network with accessible pore openings in all three crystallographic planes. This is in contrast with previously reported 2-D MOFs based on similar linear Mn_3_ SBUs but with linear terephthalic acid derivatives as the organic linkers [24,25].

**Figure 4:**
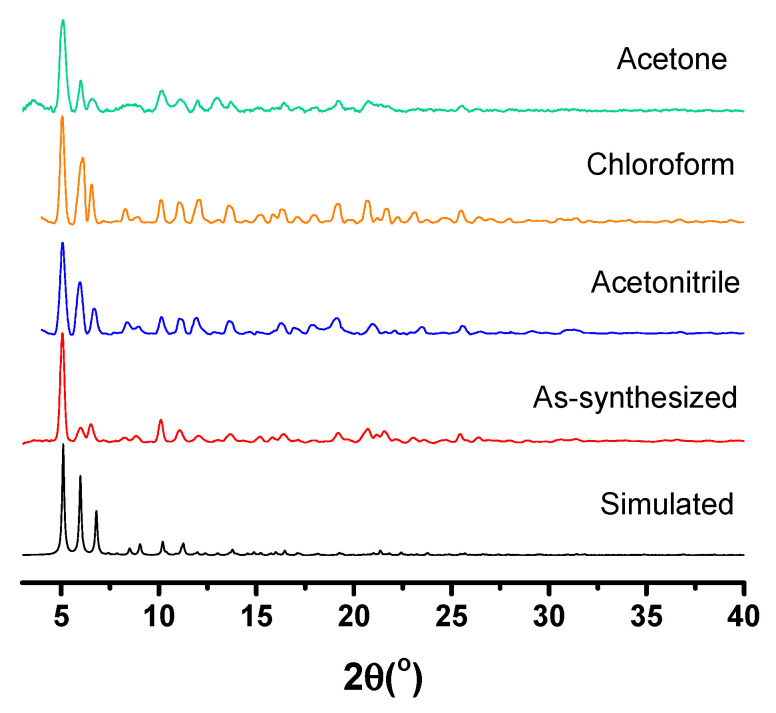
Powder X-ray diffraction patterns of simulated and as-synthesized **6**, and after solvent exchange.

The solvent stability of **6** at room temperature was assessed by suspending about 2 mg of **6** in a solvent (10 mL) for 2 weeks at room temperature. Crystals of **6** lost crystallinity and shattered slowly in common protonated solvents (methanol, ethanol, H_2_O and D_2_O) as well as in THF. However, **6** was stable to solvent exchange with acetone, chloroform and acetonitrile as shown by powder X-ray diffraction measurements (Figure 4), enabling the study of the emissions of **6** in different solvent environments. The stability of **6** at elevated temperatures was also studied by thermogravimetric analysis (TGA). The TGA of **6** (Figure S2a) exhibited a sharp mass loss from 24 °C to 50 °C, which corresponds to the loss of surface solvent. This was followed by a gradual mass loss from 63 °C to 130 °C, giving a total mass loss of 17.6%, corresponding solvent of crystallization. Material **6** was thermally stable up to 200 ^o^C, above which the onset of ligand degradation was observed. 

The bulk textural properties of **6** were then assessed by the collection of gas sorption isotherms. As-synthesized samples of **6** were activated by heating at 100 °C under a high vacuum for 8 h. Upon exposure to CO_2_, **6** showed a reversible Type-I adsorption isotherm at 196 K, the saturation capacity with a calculated BET surface area of 80 m^2^ g^−1^ (Figure S2b), indicative of limited internal microporosity. However, **6** did not adsorb N_2_, H_2_ or O_2_ probe gases to any extent, at 77 K. The low-capacity adsorption behavior of **6** can be attributed to loss of the crystalline structure upon removal of the guest solvent molecules that initially occupy the crystal pores. A powder X-ray diffraction analysis confirmed the loss of the crystallinity of **6** upon heating at 80 °C under vacuum (Figure S3). 

### 2.3. Photoluminescent Properties of ***6***

**Figure 5:**
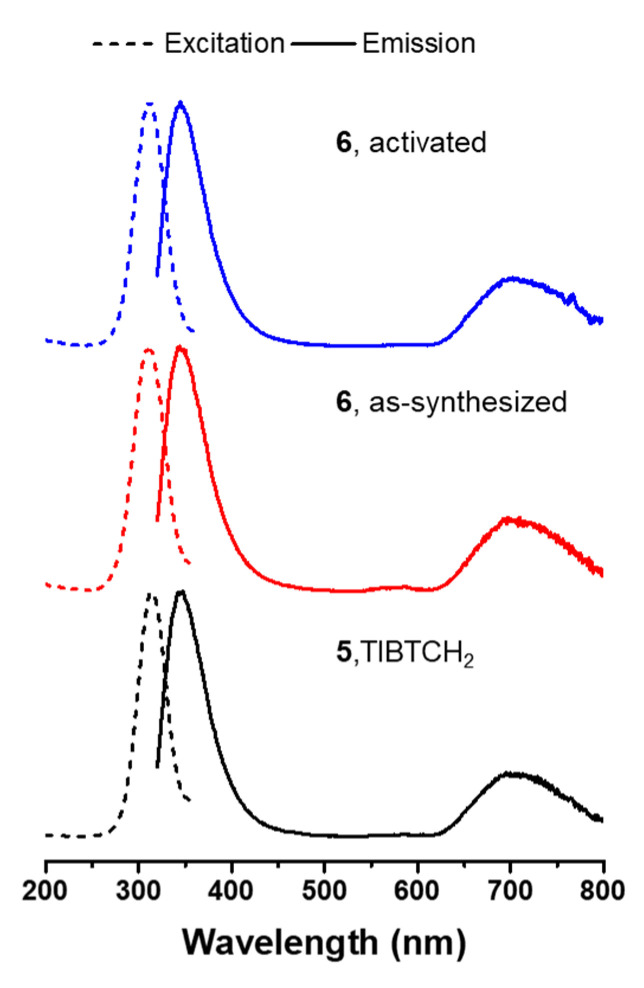
Excitation and emission profiles of activated **6**, as-synthesized **6** and **5** in a solid state.

The solid-state photoluminescence properties of **5**, as-synthesized **6,** and activated **6** were studied by fluorimetry (Figure 5). Three materials have nearly identical photophysical profiles with one excitation band near 314 nm, one sharp emission band at 345 nm, and one broad emission band at 700 nm. The data indicate that the luminescence of **6** is ligand-based. The Mn_3_ SBUs appear to have no influence on the luminescent nature of **6**, since no obvious emission quenching from LMCT was observed. The similarities of the photophysical profiles for the as-synthesized and activated **6** rules out emission from solvent molecules inside the pores. The photoluminescent properties of solvent-exchanged **6** with acetonitrile and chloroform were also studied. Material **6** possessed nearly the same excitation and emission profiles (Figure S4). There is a small hypochromic shift for the excitation (305 nm and 306 nm for acetonitrile and chloroform, respectively). The emission profile for **6** in chloroform remains unchanged, while two emission bands of **6** in acetonitrile are bathochromically shifted to 353 nm and 723 nm. This confirmed that **6** has a constant and stable photoluminescent profile under different environmental conditions. 

### 2.4. Magnetic Properties of ***6***

The solid-state magnetic properties of **6** were also assessed since the near [Mn_3_]^6+^ nodes are structurally unusual and could give rise to interesting magnetic ordering. More specifically, linear M_3_ oxo-bridged clusters are interesting model systems to study for their potential spin frustration [26]. In this case, the magnetic network of each [Mn_3_]^6+^ cluster can be simplified to have one common J parameter between Mn1 and Mn2, and it can be assumed that the single atom-O bridges will facilitate greater magnetic superexchange than the Mn-OCO-Mn bridges (see Figure 2b). The Mn(II) ions will be in the high-spin state, having a ^6^S_5/2_ ground term, and single-ion anisotropy is not expected. 

**Figure 6:**
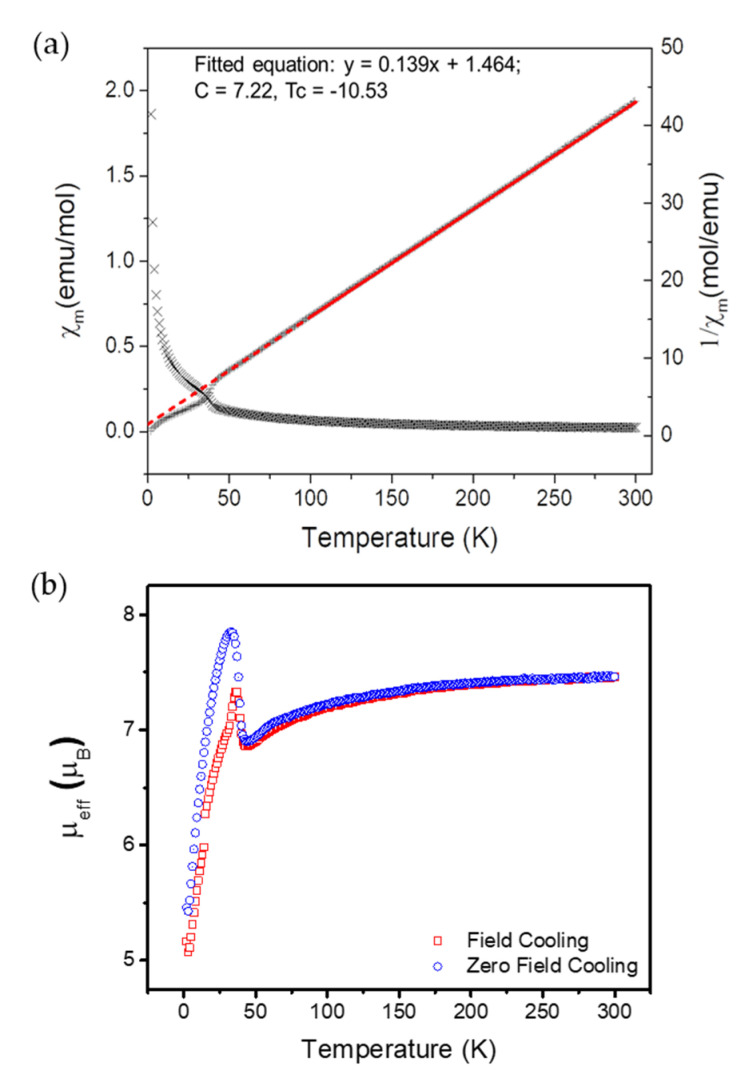
(**a**). Plot of as χ_m_ vs. T and 1/χ_m_ vs. T under the FC condition. Measured data and fitted data (100–300 K, Curie-Weiss law) are plotted in cross and red lines, respectively. The dashed red line is extrapolation of fitted data. (**b**). Plot of µ_eff_ vs. T under FC and ZFC conditions.

The magnetic properties of as-synthesized **6** were studied using a vibrating-sample magnetometer (VSM) under zero-field cooling (ZFC) and field cooling (FC) conditions from 2 K to 300 K in a 1000 Oe (G) external applied field. The temperature-dependent magnetic susceptibility and Curie–Weiss treatment of **6** under FC conditions are shown in Figure 6a. Fitting of the high temperature (100 K to 300 K) data to the Curie–Weiss law gave θ = −10.6 K, indicative of weak antiferromagnetic coupling within each Mn_3_ cluster. The corresponding value of µ_eff_ was 7.60 µ_B_ (µ_eff_ = (8C)^1/2^), which is higher than the 5.92 µ_B_ expected for an ideal S = 5/2 system.

Figure 6b shows a plot of µ_eff_ vs. temperature under both FC and ZFC conditions. At a higher temperature (>50 K), **6** exhibited nearly identical anti-ferromagnetic responses for both FC and ZFC runs. However, µ_eff_ increased below 45 K and started to differ obviously between FC and ZFC scans starting from 37 K. Under FC conditions, µ_eff_ reached its local maximum value (7.33 µ_B_) at 37 K, then decreased to 5.07 µ_B_ at 3 K. In comparison, under ZFC, µ_eff_ increased to its local maximum value (7.85 µ_B_) at 33 K and then decreased antiferromagnetically to 5.43 µ_B_ at 3 K. The nearly identical magnetic susceptibility behavior observed at low temperature in both the FC and ZFC measurements is most likely due to a phase transition from a paramagnetic (bulk disordered) phase to a more ordered phase. Based on literature precedents, this is likely caused by either the onset of long-range order or more likely due to a low-temperature phase transition that causes small distortions within the Mn_3_ clusters ca. 40 K.

We also collected magnetic hysteresis measurements at 2.0 K to further study the magnetic properties of **6** (Figure S5). The isothermal magnetization loop provided data indicative of a soft antiferromagnet with no magnetic remanence. At 9 T, **6** had still not reached saturation (4.34 emu·mol^−1^·G^–1^ for an ideal S = 5/2 system with g = 2.002). 

The data are consistent with two terminal Mn(II) atoms (Mn2) having their magnetic moments aligned anti-parallel with respect to the central Mn(II) atom (Mn1) (Figure 2b) within one linear Mn_3_ SBU. In this arrangement, the magnetic moments cancel each other, resulting in an antiferromagnetic system with an S = 5/2 ground state. Based on the distorted octahedral nature of the terminal Mn(II) ions, the actual spin vectors may not be perfectly antiparallel with respect to the central Mn1, resulting in a net magnetic susceptibility higher than an ideal S = 5/2 system. However, a complete explanation for the difference between FC and ZFC scans will only be definitively understood using other more advanced measurements (i.e., neutron diffraction analysis). One possible explanation is to attribute these magnetic moment shifts to a potential solid-state phase transition of **6** at low temperature such as a small rearrangement of the oxygen atoms around each Mn(II) atom. The change of coordination environment at low temperature may alter the net magnetism of the MOF. 

The same batch of as-synthesized **6** from VSM studies was scanned by scanning electron microscopy (SEM) equipped with the energy-dispersive X-ray spectroscopy (EDS). The SEM and EDS mapping images are shown in Figure S6. The uniform distribution of each of the elements indicated the homogeneity of the sample. No other impurities were observed. This proved that the difference between experimental and theoretical µ_eff_ values was not due to the presence of trace magnetically active impurities accompanying as-synthesized **6** polycrystals.

## 3. Conclusions

In summary, this work describes the synthesis and photophysical studies of a new type of emissive dicarboxylic acid linker that can be used to construct dual-functional MOFs with the proper choice of SBUs. We are currently investigating new strategies to construct MOFs using this new emissive linker and developing their sensing and electrochemical applications. 

## 4. Experimental Section

### 4.1. General Methods

Air- and moisture-sensitive reactions were carried out in oven-dried glassware using standard Schlenk techniques under an inert atmosphere of dry nitrogen. THF for synthesis was dried using an Innovative Technology, Pure Solv solvent purifier with a double purifying column. All the other chemicals were used from commercial sources without any further purification.

^1^H-NMR spectra were recorded on either a Mercury 400 MHz spectrometer at 400 MHz and/or a Bruker AVANCE III 500 spectrometer at 500 MHz. Coupling constants are reported in hertz (Hz), and chemical shifts are reported as parts per million (ppm) relative to residual solvent peaks residual (CD_3_)_2_SO, δH = 2.50) or tetramethylsilane (δH = 0) [27]. ^13^C NMR spectra were recorded using a Bruker AVANCE III 500 MHz NMR at 125 MHz. Chemical shifts are reported as parts per million (ppm) relative to residual solvent peaks (residual (CD_3_)_2_SO, δC = 39.52) or tetramethylsilane (δC = 0) [27]. High-resolution mass spectra (HR-MS) were obtained on an Agilent Technologies 6530 Accurate Mass Q-TOF LC/MS instrument. Infrared spectra were recorded with either a Nicolet IR 200 FTIR spectrometer or a Bruker ALPHA II’s Platinum ATR spectrophotometer. TGA analyses were performed on a TA Instruments Q50 analyzer using high-purity N_2_ carrier gas, in the range of 25–800 °C. A ramp rate of 3.50 °C s^−1^ was applied between 25 and 500 °C and 5.00 °C s^−1^ between 500–800 °C. Photoluminescence measurements were recorded on a Photon Technology International QM 4 spectrophotometer using Starna Quartz Fluorometer Cells with pathlengths of 10 mm for the solution samples and quartz tubes for solid samples. Melting points were recorded on an Electrothermal melting point apparatus in open capillaries in air and are uncorrected. UV–Vis and near-IR (NIR) absorption spectra were recorded on a Varian Cary 6000i UV–VIS–NIR Spectrophotometer Fluorimetry using Starna Quartz Fluorometer Cells with pathlengths of 10 mm. Elemental analysis was performed by Midwest Microlab LLC (Indianapolis). Gas sorption studies: the sample was activated under reduced pressure at 100 °C prior to gas uptake experiments. Gas adsorption isotherms were recorded on a Quantachrome Autosorb-1 system; all gases (99.995+%) were purchased from Praxair. Magnetization was measured with a vibrating sample magnetometer (VSM) on a physical property measurement system (PPMS) from Quantum Design with 14.65 mg as-synthesized MOF sample.

### 4.2. X-ray Diffraction

For single-crystal X-ray diffraction studies, suitable crystals were selected, covered with hydrocarbon oil and mounted on the tip of a nylon fiber on a Rigaku AFC-12 diffractometer with a Saturn 724 CCD and Rigaku XStream low-temperature device for **4** using a graphite monochromator with MoKα (λ = 0.71075 Å) and an Agilent SuperNova diffractometer with AtlasS2 CCD and an Oxford 700 low-temperature attachment for **6** using a µ-focus Cu Kα radiation source (λ = 1.5418 Å) with collimating mirror monochromators. Crystals were kept at 100 K during the data collection process. Details of crystal data, data collection, and structure refinement are listed in Table S2. Using Olex2 [28], the structure was solved with the ShelXT [29] structure solution program using Intrinsic Phasing and refined with the ShelXL [30] refinement package using Least Squares minimization. The SQUEEZE utility in PLATON [31] was applied to **6** post-refinement in order to remove residual peaks due to disordered solvent. 

For the single-crystal structure of **4**, the disorder for all thiophene groups was modeled in essentially the same way. For example, for one of the thiophene rings, the site occupancy factor for the atoms of one component of the disorder was set to the variable x. The site occupancy factors for the atoms of the alternate component were set to (1-x). The geometry of the two components was restrained to be equivalent throughout the refinement process. A common isotropic displacement parameter was refined while refining x. With the convergence of the value for x, the site occupancies were fixed, and the displacement parameters were allowed to refine while restraining their values to be both similar and approximately isotropic (SIMU and ISOR instructions).

Powder X-ray diffraction (PXRD) experiments were performed on a thin glass fiber using perfluoropolyether oil in a Rigaku R-Axis Spider diffractometer using CuKα radiation with data collected in the range 5–40° 2θ. Simulated PXRD was generated using single-crystal reflection data via SimPowPatt facility in PLATON.

### 4.3. Compound Synthesis

**5**, (5,5’-(1H-thieno[3,4-d]imidazole-4,6-diyl)bis(thiophene-2-carboxylic acid), (TIBTCH_2_): **4** [19] (2.03 g, 7.04 mmol) was dissolved in dry THF (80 mL) in an oven-dried 200 mL Schlenk flask fitted with a 125 mL addition funnel. N-BuLi (1.6 M in hexanes, 25.6 mmol) was added dropwise through the addition funnel to the vigorously stirred solution at −78 °C over a period of 1 h. The addition funnel was then removed, and the solution was stirred at −78 °C (2 h) before being allowed to slowly warm to room temperature over a period of 20 min. The mixture was then stirred at room temperature (10 min) before being recooled to −78 °C. Ground solid CO_2_ (approx. 100 g) was then added to fill the Schlenk flask under a stream of N_2_. Stirring was continued while the temperature was allowed to rise to room temperature (8 h). The mixture was filtered and a red solid was collected. The solid was re-dissolved in DI water (500 mL). After washing with Et_2_O, CH_2_Cl_2_ and EtOAc (100 mL ea.), the aqueous phase was acidified with conc. HCl to pH < 2 (pH paper) resulting in the precipitation of a dark red solid. The precipitate was collected by filtration under reduced pressure and then suspended in warm acetone (200 mL), and the mixture was stirred at room temperature (8 h). The mixture was then collected by filtration and further washed with acetone (50 mL × 3) to collect 2.58 g of a dark red sold (97%); ^1^H-NMR (500 MHz, (CD_3_)_2_SO) δ 8.59 (s, 1H), 7.71 (d, *J* = 4.0 Hz, 2H), 7.50 (d, *J* = 4.0 Hz, 2H); ^13^C NMR (126 MHz, (CD_3_)_2_SO) δ 162.75, 153.78, 141.03, 140.47, 134.60, 132.33, 124.78, 109.84.; FT-IR (cm^−1^): 2779 (br, w), 1652 (s), 1521 (m), 1487 (m), 1435 (s), 1377 (m), 1344 (w), 1275 (m), 1225 (s), 1201 (m), 1099 (m), 1033 (m), 986 (m), 940 (w), 904 (w), 893 (w), 862 (w), 801 (m), 786 (m), 742 (s), 699 (w), 646 (m), 603 (w), 576 (m), 514 (s). HR-MS (ESI+): [M + H]^+^ (C_15_H_9_N_2_O_4_S_3_^+^), calcd. 376.9719, found: 376.9728. m.p.: 205–208 °C.

**6**, (2H·[Mn_3_·(TIBTC)_4_]·3H_2_O): **5** (15.0 mg, 0.04 mmol) and Mn(OAc)_2_·2H_2_O (25.5 mg, 0.10 mmol) were suspended in a mixture of H_2_O and *N*,*N*-dimethylformamide (10% water in N,N-dimethylformamide) in a 20 mL scintillation vial, which was surpersonicated for 20 min before heating by submersion in a pre-heated graphite thermal bath and held at 85 °C for 7–10 days. The crystals of **6** were isolated by washing and decanting any impurities away using a 10% water in *N*,*N*-dimethylformamide mixture. The solid was then filtered using a Büchner funnel and thoroughly washed with the same solvent. The crystalline solid was dried in the air. Average yield, 3.2 mg (9.2%, calculated from 4). FT-IR (cm^−1^): as-synthesized: 3062 (w), 2071 (m), 2021 (w), 1994 (w),1871 (s), 1675 br/(m), 1585 (w), 1477 (m), 1432 (m), 1306 (w), 1260 (w), 1183 (w), 1158 (w), 1088 (m), 1068 (w), 1025 (m), 998 (w), 801 (w), 740 (s), 691 (s), 624 (m), 609 (m), 573 (s), 509 (s), 493 (s), 446 (m), 416 (s). Activated: 3482 (w), 3275 (m), 3237 (m), 3157 (m), 2955 (m), 2929 (m), 2881 (m), 1641 (w), 1584 (m), 1522 (br,w), 1483 (m), 1438 (m), 1356 (m), 1328 (m), 1274 (m), 1152 (m), 1100 (m), 1015 (s), 978 (m), 905 (w), 764 (m), 655 (s), 583 (w), 516 (s), 490 (s), 447 (m). Elemental analysis (C_60_H_32_Mn_3_N_8_O_19_S_12_): Calcd. C, 41.94%; H, 1.88%; N, 6.52%; S, 22.39%. Found. C, 41.96%; H, 1.41%; N, 6.52%; S, 22.46%.

## Data Availability

Not applicable.

## Supplementary Materials

The following are available online. Table S1. Bond valance sum (BVS) method for Mn1 and Mn2 in **6**. Figure S1. Deconvoluted Mn 2s spectra of as-synthesized **6** polycrystals. Figure S2. (a). TGA profile of as-synthesized **6**, obtained at a heating rate of 3.5 °C from 23 °C to 500 °C and a heating rate of 5 °C from 500 °C to 800 °C. (b) Adsorption isotherms of carbon dioxide for **6**. Figure S3. Powder X-ray diffraction analysis of **6** heating at 80 °C under vacuum. Figure S4. Excitation (red) and emission (blue) profiles of solvent-exchanged and as-synthesized **6** polycrystalline sample. Figure S5. Magnetic hysteresis curve of **6** (as-synthesized) at 2 K. Figure S6. The SEM image (a) and EDS spectra of S (b), Mn (c). and O (d) signal of as-synthesized **6**. Table S2. Crystal data and structure refinement for **4** and **6**, spectroscopic images for all new compounds (^1^H NMR, ^13^C NMR) and text tables of crystallographic data, S1. 1H-NMR of **4**, S2. 13C-NMR of **4**, S3. 1H-NMR of **5**, S4. 13C-NMR of **5**.
