# Supplementary material for: Magnetism and Luminescence of a MOF with Linear Mn3 Nodes Derived from an Emissive Terthiophene-Based Imidazole Linker
Source: Molecules. 2021 Jul 15;26(14):4286. doi: 10.3390/molecules26144286 (PMC8305848; doi:10.3390/molecules26144286)

Magnetism and luminescence of a MOF with linear Mn_3 nodes derived from an emissive terthiophene-based imidazole linker.

Weiran Wang, Junpeng He, Hongyu Guo, Samuel G. Dunning, Simon M. Humphrey and Richard A. Jones **

Department of Chemistry, The University of Texas at Austin, 105 E. 24th Street, Stop A5300, Austin, Texas 78712-1224, USA.

Supporting Information

Table of Contents

Table S1. Bond valance sum (BVS) method for Mn1 and Mn2 in **6**. 3

Fig. S1. Deconvoluted Mn 2s spectra of as-synthesized **6** polycrystals. 4

Fig. S2. (a). TGA profile of as-synthesized **6**, obtained at a heating rate of 3.5 ºC from 23 ºC to 500 ºC and a heating rate of 5 ºC from 500 ºC to 800 ºC. (b) adsorption isotherms of carbon dioxide for **6**. 5

Fig. S3. Powder X-ray diffraction analysis of **6** heating at 100 ºC under vacuum. 6

Fig. S4. Excitation (red) and emission (blue) profiles of solvent-exchanged and as-synthesized **6** poly crystalline sample. 7

Fig. S5. Magnetic hysteresis curve of **6** (as-synthesized) at 2 K. 8

Fig. S6. The SEM image (a) and EDS spectra of S (b), Mn (c) and O (d) signal of as-synthesized **6**. 9

Table S2. Crystal data and structure refinement for **4** and **6**. 10

S1. ^{1}H-NMR of **4** 122

S2. ^{13}C-NMR of **4**. 133

S3. ^{1}H-NMR of **5**. 144

S4. ^{13}C-NMR of **5**. 155

Table S1. Bond valance sum (BVS) method for Mn1 and Mn2 in **6**.

	Mn1*			
	Bond Distance (R_{ij}, Å)	s(Mn(II))	s(Mn(III))	s(Mn(IV))
O1	2.177	0.351	0.239	0.319
O4	2.192	0.338	0.224	0.307
O5	2.129	0.393	0.290	0.363
Total bond valence**		2.165	1.505	1.978
	Mn2*			
	Bond Distance	s(Mn(II))	s(Mn(III))	s(Mn(IV))
O2	2.100	0.422	0.326	0.392
O3	2.332	0.242	0.127	0.211
O4	2.242	0.300	0.183	0.268
O6	2.082	0.440	0.350	0.412
O7	2.234	0.306	0.189	0.274
O8	2.182	0.346	0.234	0.315
Total bond valence		2.056	1.410	1.872

* Parameter used [21]: Mn(II): R_0 = 1.740, B_0 = 0.417; Mn(III): R_0 = 1.823, B_0 = 0.247; Mn(III): R_0 = 1.750, B_0 = 0.374. s(Mn(n)) = $\exp[(R_0 - R_{ij})/B_0]$.

** Due to the octahedral nature of Mn1, each of O1, O4 and O5 were counted twice.

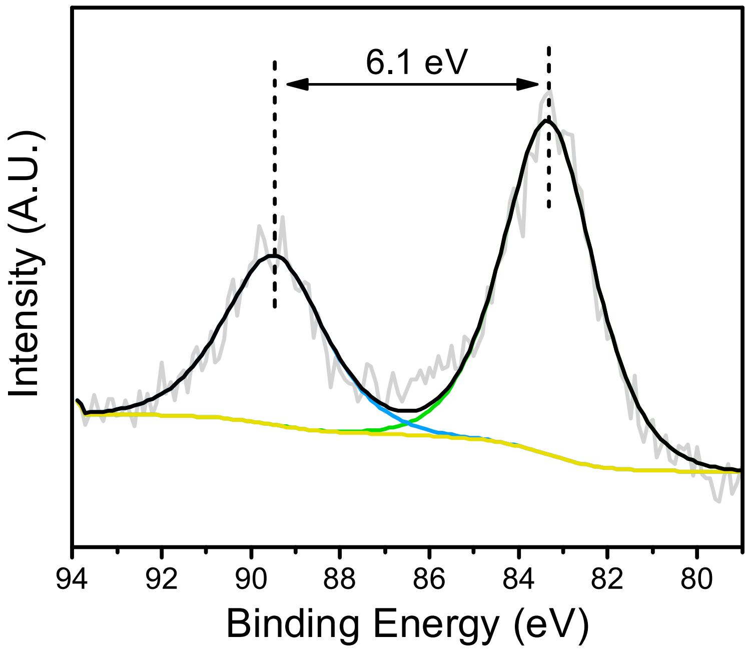

Fig. S1. Deconvoluted Mn 2s spectra of as-synthesized **6** polycrystals.

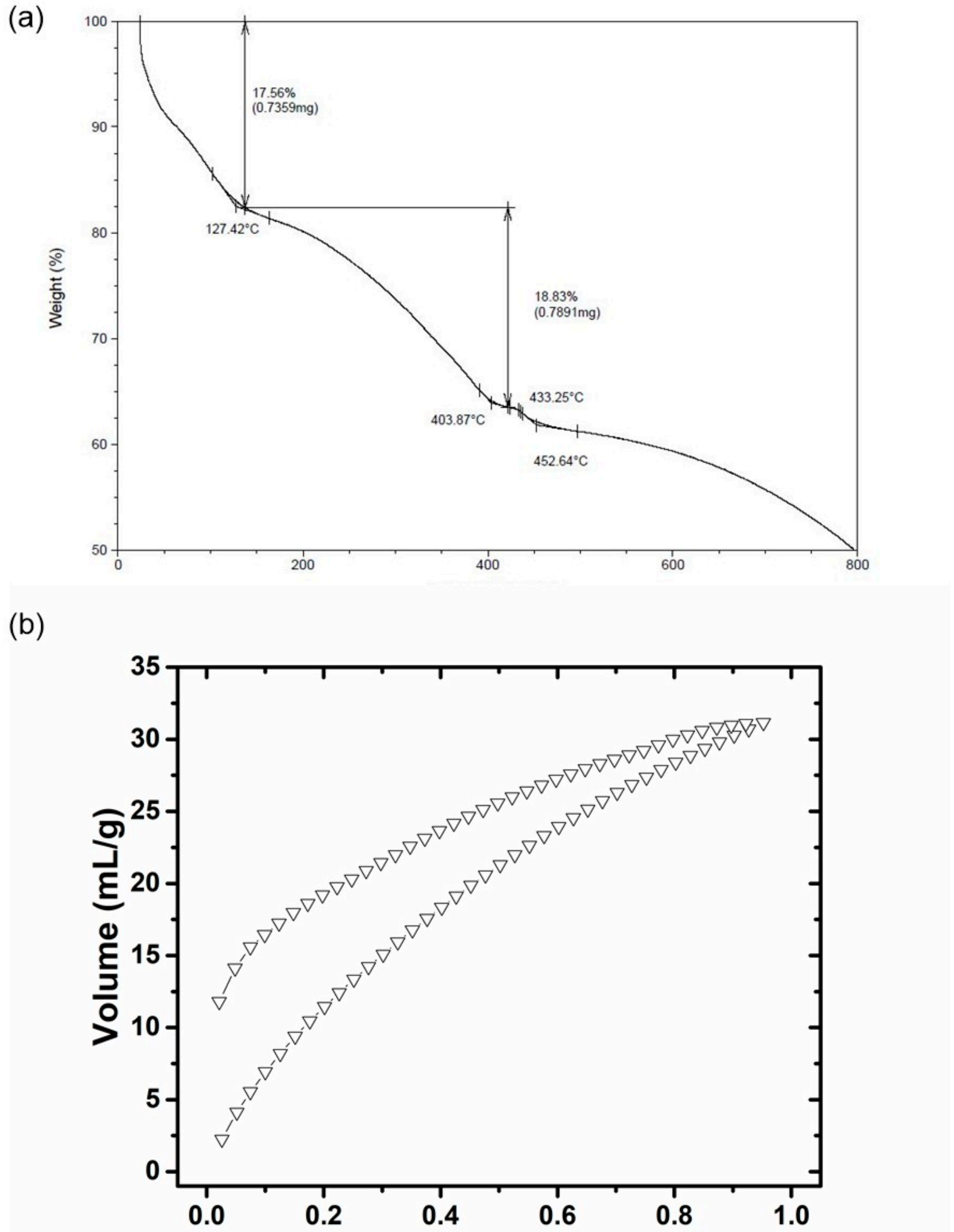

Fig. S2. (a). TGA profile of as-synthesized **6**, obtained at a heating rate of 3.5 ºC from 23 ºC to 500 ºC and a heating rate of 5 ºC from 500 ºC to 800 ºC. (b) adsorption isotherms of carbon dioxide for **6**.

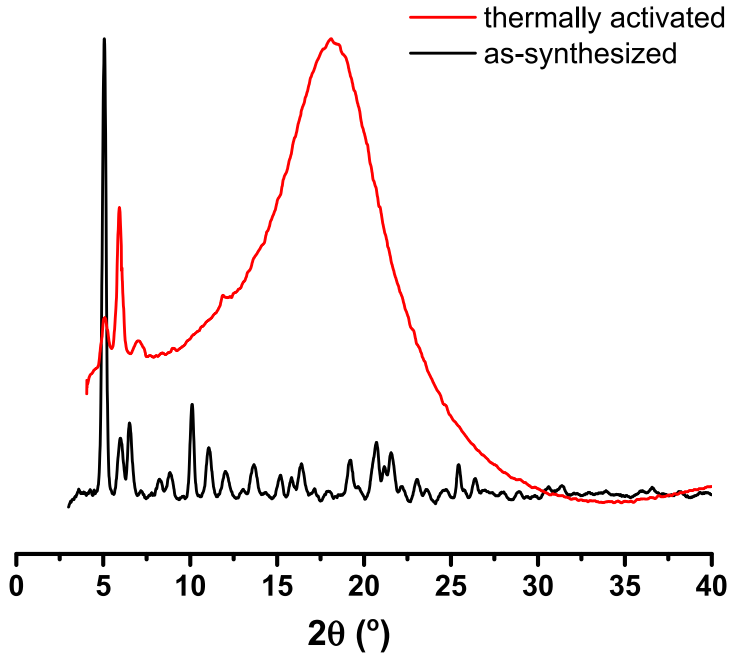

Fig. S3. Powder X-ray diffraction analysis of **6** heating at 100 ºC under vacuum.

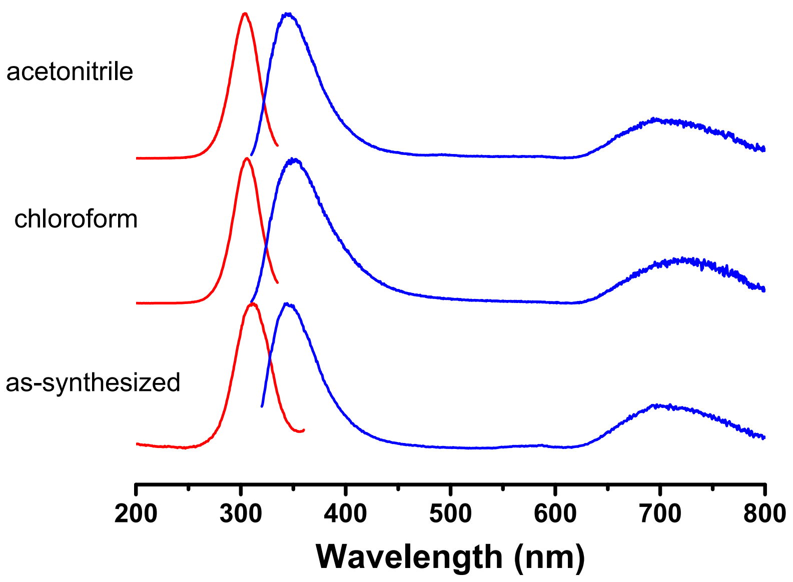

Fig. S4. Excitation (red) and emission (blue) profiles of solvent-exchanged and as-synthesized **6** poly crystalline sample.

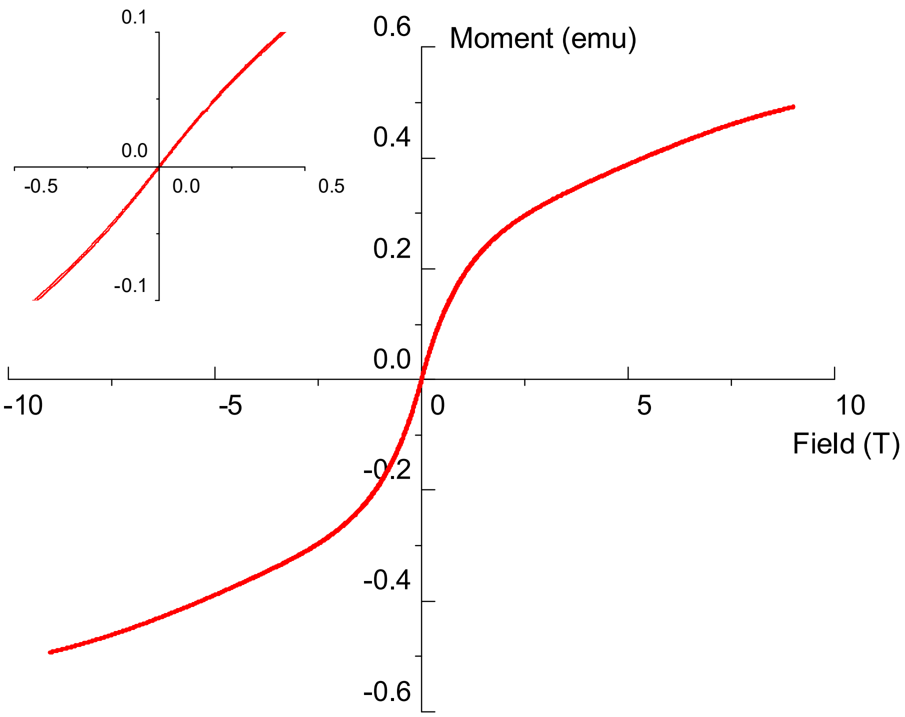

Fig. S5. Magnetic hysteresis curve of **6** (as-synthesized) at 2 K.

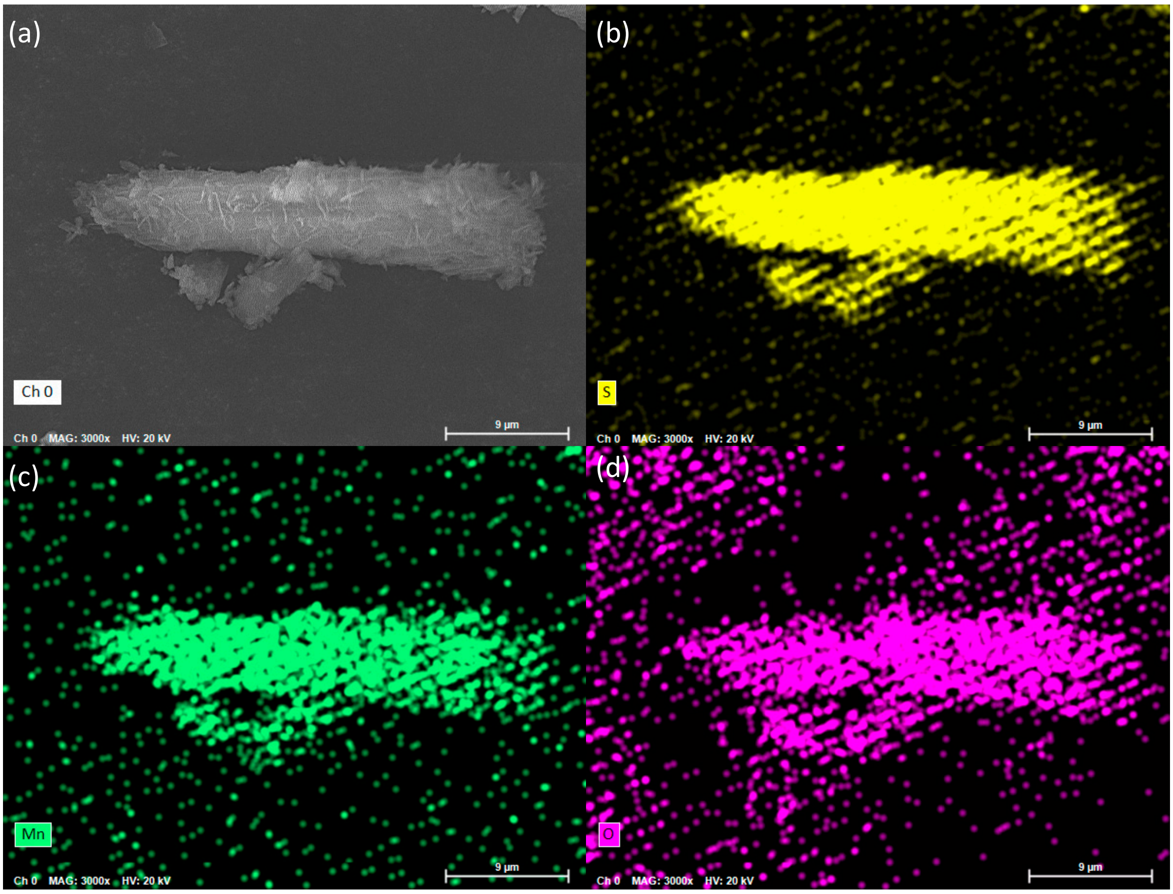

Fig. S6. The SEM image (a) and EDS spectra of S (b), Mn (c) and O (d) signal of as-synthesized **6**.

Table S2. Crystal data and structure refinement for **4** and **6**.

Identification code	**4-DTTI**	**6**
Empirical formula	$C_{14.75}H_{10}N_2S_3$	$C_{30}H_{13}Mn_{1.5}N_4O_8S_6$
Formula weight	311.43	832.21
Temperature/K	100.15	100.01(11)
Crystal system	monoclinic	orthorhombic
Space group	$P2_1/n$	*Pcca*
a/Å	9.2726(11)	29.5736(10)
b/Å	13.1237(16)	17.3377(8)
c/Å	23.051(3)	25.9995(9)
α/°	90	90
β/°	90.328(3)	90
γ/°	90	90
Volume/Å^3	2805.1(6)	13330.9(9)
Z	8	8
ρ_{calc} g/cm^3	1.475	0.829
μ/mm^{-1}	0.517	4.354
F(000)	1284	3348
Crystal size/mm^3	0.24 × 0.09 × 0.06	0.188 × 0.071 × 0.05
Radiation	MoKα (λ = 0.71075 Å)	CuKα (λ = 1.54184 Å)
2Θ range for data collection/°	4.704 to 54.966	9.012 to 136.476

Index ranges<br><br>Reflections collected	$-12 \leq h \leq 12$, $-17 \leq k \leq 17$, $-29 \leq l \leq 29$<br><br>43111	$-20 \leq h \leq 35$, $-15 \leq k \leq 20$, $-31 \leq l \leq 21$<br><br>38321
Independent reflections	6413 [R_{int} = 0.0718, R_{sigma} = 0.0520]	12184 [R_{int} = 0.1188, R_{sigma} = 0.1263]
Data/restraints/parameters	6413/1231/544	12184/300/448
Goodness-of-fit on F^2	1.057	0.944
Final R indexes [I>=2σ (I)]	R_1 = 0.0536, wR_2 = 0.0992	R_1 = 0.0813, wR_2 = 0.2098
Final R indexes [all data]	R_1 = 0.0804, wR_2 = 0.1086	R_1 = 0.1294, wR_2 = 0.2421
Largest diff. peak/hole / e $Å^{-3}$	0.34/-0.34	1.08/-0.66

S1. ^{1}H-NMR of **4**.

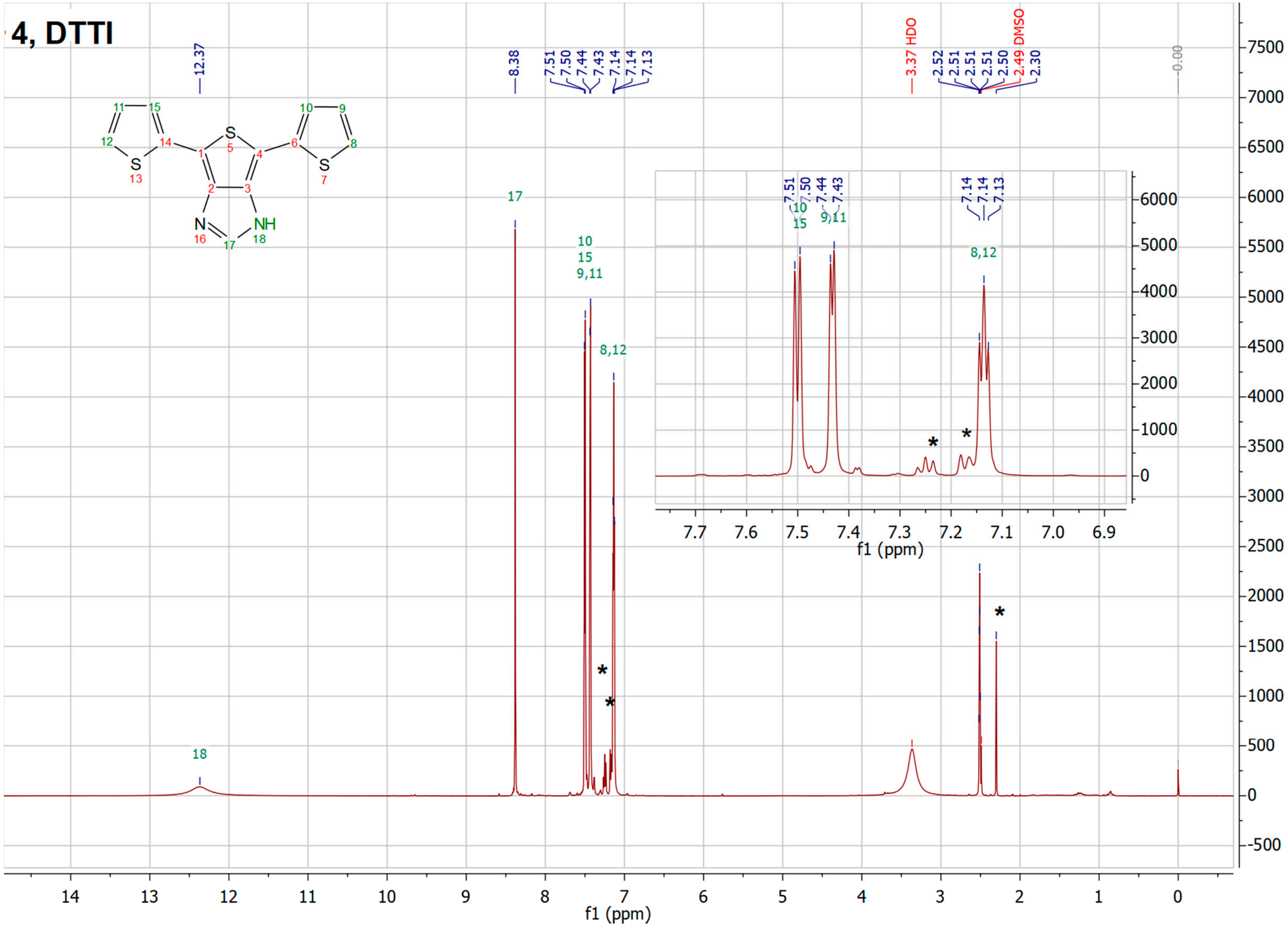
4, DTTI
12.37
8.38
7.51
7.50
7.44
7.43
7.14
7.14
7.13
3.37 HDO
2.52
2.51
2.51
2.51
2.50
2.49 DMSO
2.30
0.00
17
10
15
9,11
8,12
18
*
f1 (ppm)

S2. ^{13}C-NMR of **4**.

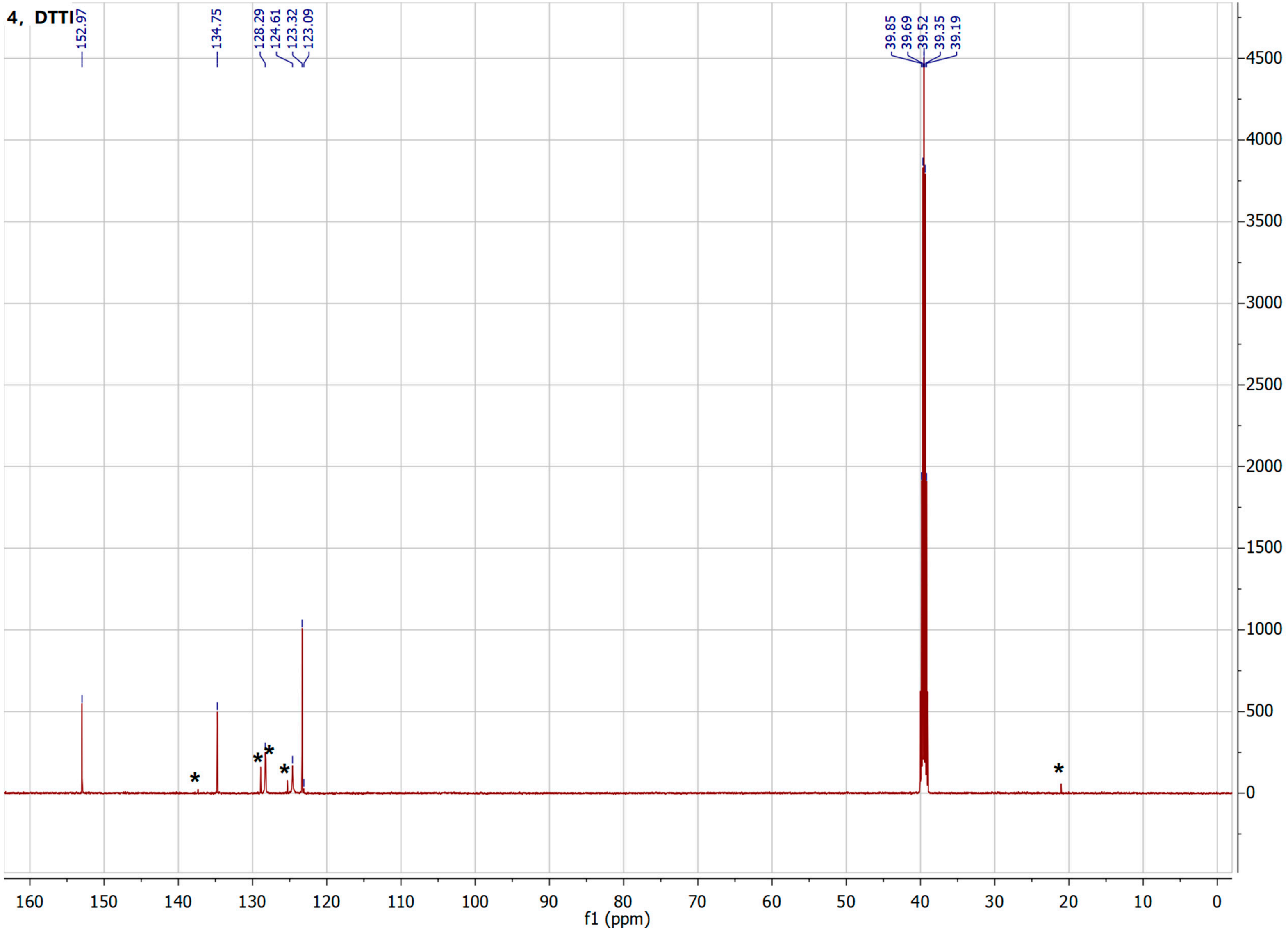

S3. ^{1}H-NMR of **5**.

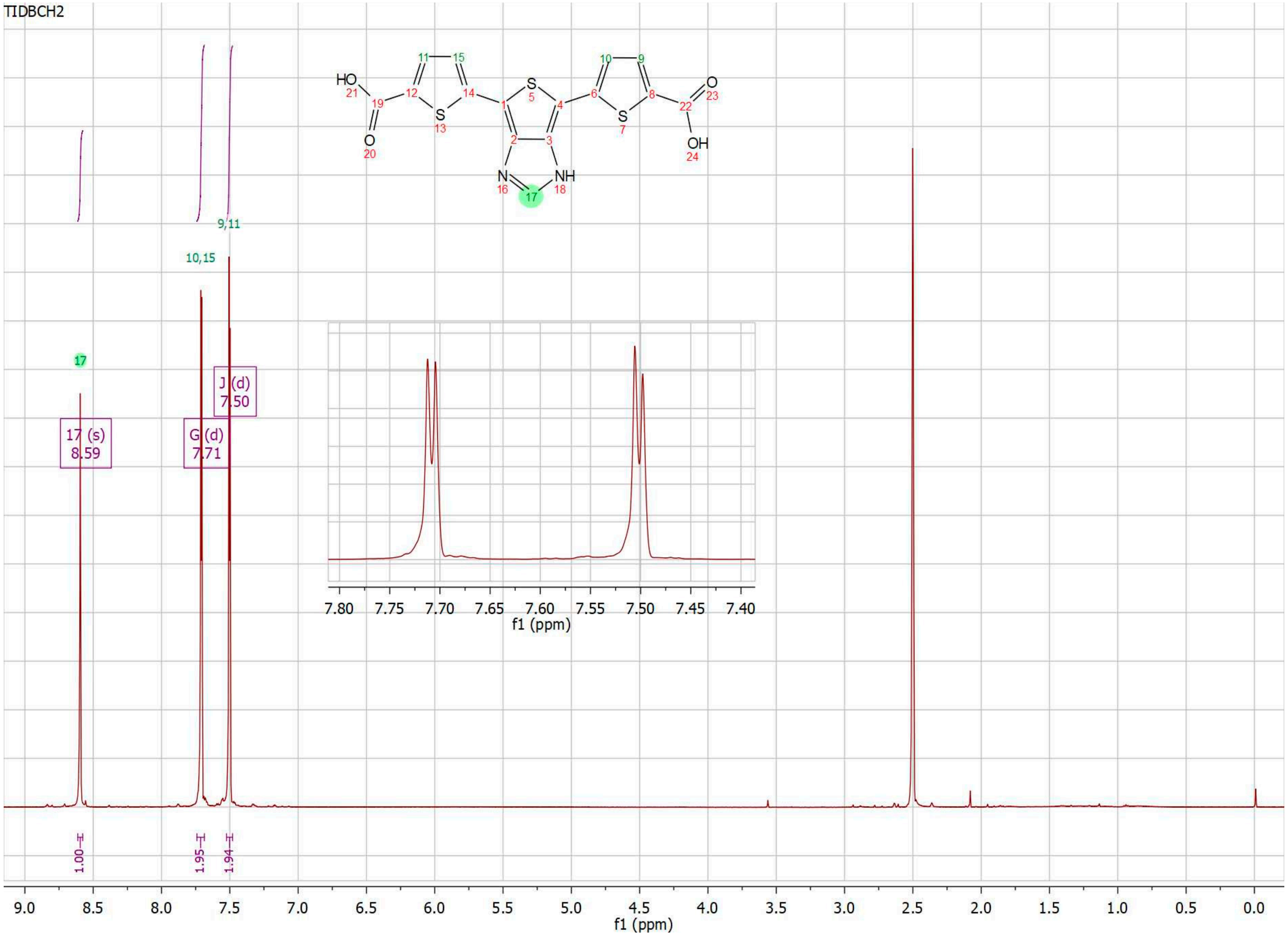
TIDBCH2
HO
21
19
12
11
15
14
S
13
O
20
1
S
5
4
2
3
N
16
17
NH
18
6
10
9
8
S
7
22
O
23
OH
24
9,11
10,15
17
J (d)
7.50
17 (s)
8.59
G (d)
7.71
7.80
7.75
7.70
7.65
7.60
7.55
7.50
7.45
7.40
f1 (ppm)
1.00
1.95
1.94
9.0
8.5
8.0
7.5
7.0
6.5
6.0
5.5
5.0
4.5
4.0
3.5
3.0
2.5
2.0
1.5
1.0
0.5
0.0
f1 (ppm)

S4. ^{13}C-NMR of **5**.

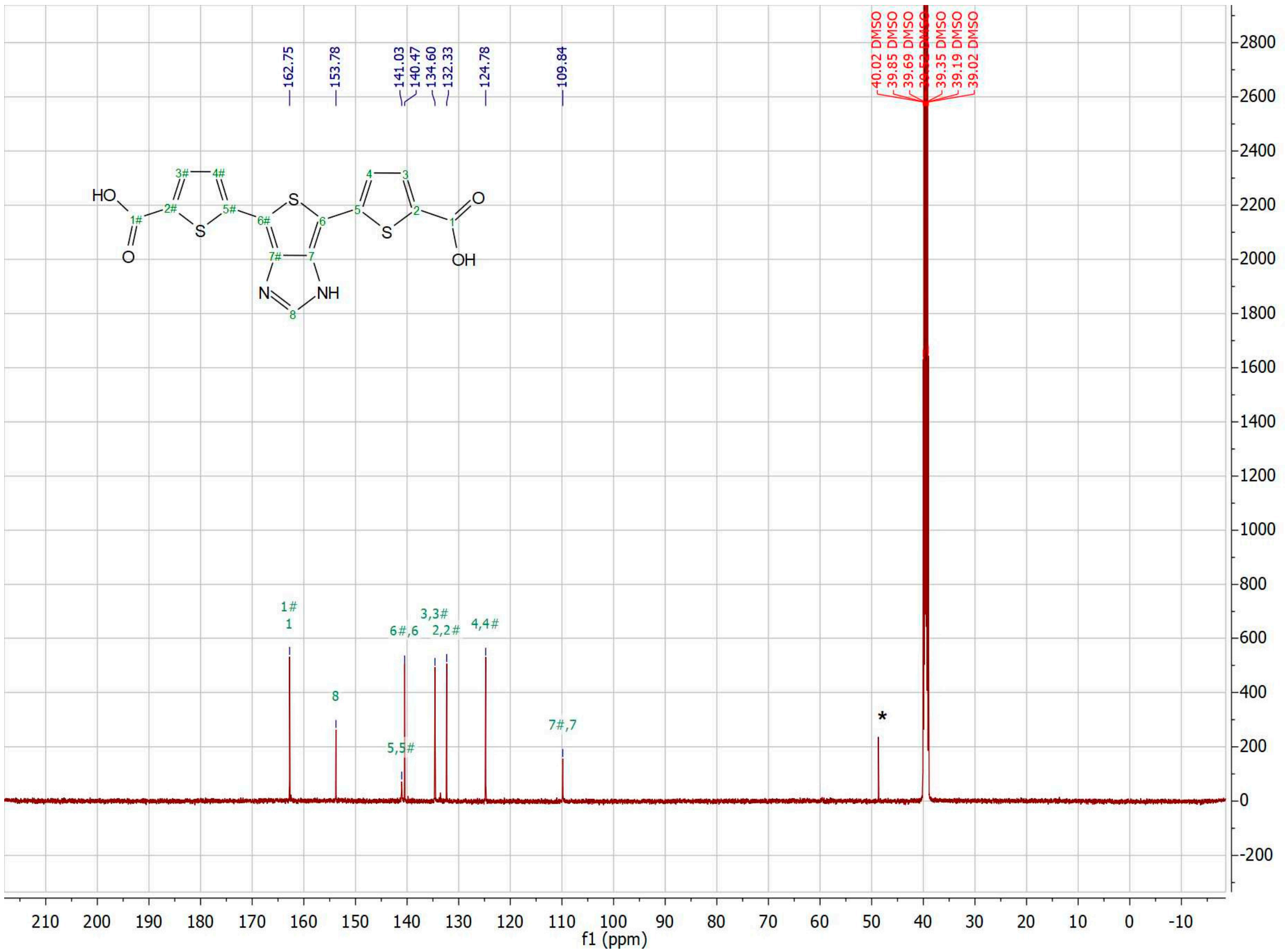

Supplement: Supplementary file 1 [file molecules-26-04286-s001.zip › molecules-1266478-supplementary.pdf]
